# Adiponectin and Orexin-A as a Potential Immunity Link Between Adipose Tissue and Central Nervous System

**DOI:** 10.3389/fphys.2018.00982

**Published:** 2018-07-24

**Authors:** Rita Polito, Ersilia Nigro, Antonietta Messina, Maria L. Monaco, Vincenzo Monda, Olga Scudiero, Giuseppe Cibelli, Anna Valenzano, Elisabetta Picciocchi, Christian Zammit, Daniela Pisanelli, Marcellino Monda, Ivan R. Cincione, Aurora Daniele, Giovanni Messina

**Affiliations:** ^1^Dipartimento di Scienze e Tecnologie Ambientali Biologiche e Farmaceutiche, Università degli Studi della Campania “Luigi Vanvitelli”, Caserta, Italy; ^2^Dipartimento di Scienze Cardio-Toraciche e Respiratorie, Università degli Studi della Campania “Luigi Vanvitelli”, Naples, Italy; ^3^Sezione di Fisiologia Umana e Unità di Dietetica e Medicina dello Sport, Dipartimento di Medicina Sperimentale, Università degli Studi della Campania “Luigi Vanvitelli”, Naples, Italy; ^4^CEINGE-Biotecnologie Avanzate s.c. a r.l., Naples, Italy; ^5^Dipartimento di Medicina Molecolare e Biotecnologie Mediche, Università degli Studi di Napoli Federico II, Naples, Italy; ^6^Department of Clinical and Experimental Medicine, University of Foggia, Foggia, Italy; ^7^Department of Anatomy, University of Malta, Msida, Malta

**Keywords:** adiponectin, orexin-A, immunity, adipose tissue, central nervous system

## Abstract

Adipose tissue (AT) is strongly associated with development and progression of immune disorders through adipokines secretion, such as adiponectin. This protein has beneficial energetic properties and is involved in inflammation and immunity processes. Three oligomers of circulating adiponectin with different molecular weight are described: High (HMW), Medium (MMW), and Low (LMW). The HMW is the most biologically active oligomers. On binding to its receptors AdipoR1, AdipoR2, and T-cadherin, adiponectin acts on both innate and acquired immunity. The suppression of NF-κB activation and pro-inflammatory cytokine expression in macrophages is mediated by AdipoR1. AdipoR2 mediates polarization of anti-inflammatory M2 macrophages T-cadherin is essential for the M2 macrophage proliferation. Furthermore, adiponectin reduces T cells responsiveness and B cells lymphopoiesis. The immune system is very sensitive to environmental changes and it is not only interconnected with AT but also with the central nervous system (CNS). Cytokines, which are mediators of the immune system, exercise control over mediators of the CNS. Microglia, which are immunity cells belonging to the macrophage family, are present within the CNS. The nervous system is also involved in immunity through the production of neuropeptides such as orexin-A/hypocretin-1. This neuropeptide is involved in metabolic disorders, inflammation and in the immune response. The relationship between adipokines, immunity, and the nervous system is validated by both the role of orexin-A on fat, food intake, and energy expenditure, as well as by role of adiponectin on the CNS. In this review, we focused on the functions of adiponectin and orexin-A as a potential immunity link between AT and CNS.

## Introduction

Adipose tissue (AT) is a multifunctional organ involved in many physiological and metabolic processes. Its functions are both as a site for energy storage and as an endocrine organ, fundamentally composed of adipocytes but populated also by a number of immune cells such as T lymphocytes and macrophages (Coelho et al., 2013). As a result of the excessive expansion of AT mass, activation of lipolysis, eating a high fat diet, and even non-shivering thermogenesis, results in numerous immune cells being recruited in AT and activated (Ferrante, 2013). Through the production of adipokines and in particular of adiponectin, AT is involved in many metabolic functions such as energy reserve, and thermoregulation. In addition, the literature data demonstrated that obese people have a higher incidence of immune and autoimmune diseases, such as common variable immunodeficiency, rheumatoid arthritis, and multiple sclerosis (Osborn and Olefsky, 2012). In pathological and non-pathological situations the immune system monitors and responds to specific metabolic signals. This is done via a set of factors such as adipocytokines that have important roles in many physiological and pathological processes, like immune responses. On the other hand, the immune system is very sensitive to environmental changes (Paparo et al., 2014; Sessa et al., 2018b). Nutrition constitutes one of the major environmental factors. It exerts several effects on the function and development of the immune system. Thus, it influences one’s health and the risk to develop diseases. In particular, nutrient excesses or deficiencies alter immunocompetence (Ingvartsen and Moyes, 2013). Undernutrition depresses immune system. Similarly, excessive intake of nutrients and vitamins reduces the immune competence. It is well known that the AT is pervaded by cells of the immune system. Obesity induced a radical change in the immune cells that populate this organ. The immune system also communicates with the central nervous system (CNS) through different mediators. The CNS is involved in energy homeostasis, food intake, and metabolic processes through the production of neuropeptides such as orexin-A/hypocretin-1 (Chieffi et al., 2017). Due to the connection between adipokines, immunity, and the nervous system, this review will be focused on the role of adiponectin and orexin A in immunity.

## Adipose Tissue in Immunity

The AT is the main deposition of triglycerides in mammals, including man. One of the main functions of adipocytes is the synthesis and release of triglycerides. The number of cells and the dimension of adipose mass depend on the nutrition state of the individual. Excessive caloric intake (obesity) results in an increase in adipose mass, while chronic nutritional deficiencies result in the opposite outcome. It is well known that AT is constituted not only by adipocytes but by a plethora of different immune cells, such B cells, T cells, as macrophages, and dendritic cells that produce anti-inflammatory cytokines (Brestoff et al., 2015; Salerno et al., 2018). In a chronic inflammatory state, such as in obesity, the cells present in AT undergo a radical change. There is also an alteration in the production of adipokines, hormones produced and released by adipocytes (Makki et al., 2013). Adipokines are involved in several metabolic and inflammatory processes as well as in the normal homeostasis of many organs and tissues. To date, many adipokines have been identified. Some of them, such as leptin, resistin, visfatin, chemerin, and adipsin, have pro-inflammatory effects. Leptin and visfatin, in particular, act directly on immune cells. They increase neutrophil recruitment, macrophages and NK cells activation, lymphocytes chemotaxis, T cells activation, and decrease Treg recruitment. On the other hand, some other adipokines, such as adiponectin and omentin, are involved in anti-inflammatory processes. They reduce T cells responsiveness, B cells lymphopoiesis, monocyte adhesion, TLR4 activation, and pro-inflammatory mediators such as TNF-α while increasing the production of IL-10 (Ghigliotti et al., 2014).

## Adiponectin in Immunity

**FIGURE 1:**
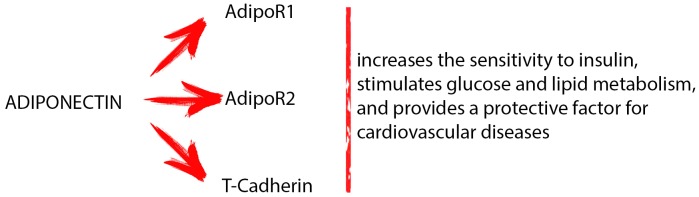
Through different receptors (AdipoR1, AdipoR2, and T-cadherin), adiponectin exerts various functions: it increases the sensitivity to insulin, stimulates glucose and lipid metabolism, and provides a protective factor for cardiovascular diseases.

Adiponectin is the most abundant adipokine produced by AT. This protein can be found in three different molecular weight: high (HMW), medium (MMW), and low molecular weight (LMW). The HMW adiponectin oligomers are the most biologically active. Adiponectin acts by binding two membrane specific receptors located in numerous organs and tissues: AdipoR1 and AdipoR2. These receptors belong to the seven-transmembrane receptor family coupled with G proteins and possess the N-terminal domain within the cell and the C-terminal outside. AdipoR1 and AdipoR2 differ in the specificity of binding to the adiponectin. AdipoR1 is activated both by the full-length as well as by the globular form o adiponectin. Instead, AdipoR2 has more affinity for the full-length form. A third receptor of the adiponectin is T-cadherin. The latter is a calcium-dependent adherence molecule that presents the classical cadherin structure, even if it does not have a cytoplasmic or transmembrane domain. T-cadherin is widespread ubiquitous, with high expression in the cardiovascular system and low levels in the muscles. T-cadherin recognizes only the MMW and HMW oligomers (Daniele et al., 2012). Through AdipoR1, AdipoR2, and T-cadherin, adiponectin exerts various functions (**Figure 1**). It increases the sensitivity to insulin, stimulates glucose, and lipid metabolism, and provides a protective factor for cardiovascular diseases (Balsan et al., 2015; Sessa et al., 2018a). Interestingly, adiponectin receptors are presents on the surface of many immune cells. This results in adiponectin having different effects on the cells of innate and acquired immunity. It is well known that AdipoR1, AdipoR2, and T-cadherin are present on macrophages. The binding of adiponectin on the AdipoR1 and AdipoR2 present on these cells results in their proliferation and polarization (Hutcheson, 2015). On the other hand, the suppression of NF-κB activation and pro-inflammatory cytokine expression in macrophages is mediated by AdipoR1; AdipoR2 mediates polarization of anti-inflammatory M2 macrophages, while T-cadherin is very important, stimulating the M2 macrophage proliferation through the adiponectin action (Chinetti et al., 2004; Shih et al., 2015). As reported by Hui et al. (2015), there is a strong link between adiponectin and immune cells such as macrophages. Adiponectin enhances cold-induced browning of subcutaneous AT through M2 macrophage proliferation. On binding to the T-cadherin present on the surface of M2 macrophages, adiponectin promotes cell proliferation by activation of Akt, consequently leading to beige cell activation (Hui et al., 2015). Adiponectin acts also on monocytes and suppresses the transcriptions of TNF-α, IL-6, and IL-8 in monocytes and macrophages (Awazawa et al., 2011). A pivot role in the regulation of non-macrophage innate immune cells is played by adiponectin: through common or different intracellular signaling pathways it can suppress the activation of eosinophils, neutrophils, T cells, NK cells, and DCs (Luo et al., 2011; Spahn et al., 2014). Moreover, Tsuchida et al. (2005) suggested that adiponectin through innate immune response-dependent mechanisms can regulate insulin sensitivity and energy expenditure. In addition, adiponectin acts on acquired immune cells reducing T cells responsiveness and B cells lymphopoiesis (Lago et al., 2007). On the other hand, different studies demonstrated a different role of adiponectin in immune and autoimmune diseases (Peters et al., 2010). The literature data reported that adiponectin serum levels increase in autoimmune diseases and decrease in immunodeficiency diseases (Toussirot et al., 2012). Hietaharju et al. (2010) reported that adiponectin levels in cerebrospinal fluid of multiple sclerosis patients are significantly higher when compared to controls subjects. Chen et al. (2013) reported that adiponectin levels in serum and in synovial fluid of rheumatoid arthritis patients are much higher when compared to controls. In addition, *in vitro* study on chondrocytes suggested that adiponectin promotes rheumatoid arthritis (Lee et al., 2008). On the contrary, Pecoraro et al. (2017) reported that adiponectin serum levels in patients affected by common variable immunodeficiency are much lower when compared to controls subjects. Although the fact that adiponectin influences the immune system is well accepted, the underlying molecular mechanisms by which it acts are not yet clear. Some studies suggest that the different modulation of adiponectin in these diseases could also be attributable to the different oligomers that are involved and/or to the presence of different and specific receptors on target cells (Wilk et al., 2013).

These studies suggest a controversial role for adiponectin plasma levels and inflammation. Summarily, it could be suggested pro-inflammatory effects in classic chronic inflammatory/autoimmune disease and an anti-inflammatory action in obesity patient.

## Central Nervous System in Immune Response

The nervous system plays important functions monitoring and coordinating organ function and responding to changes in the external environment. This system can be divided into two parts, the CNS, and the peripheral nervous system, and includes the brain, spinal cord, and a complex network of neurons. The CNS has involved in different processes through the production of neuropeptides such as orexin-A/hypocretin-1 (Black, 1994). Recently, the involvement of the CNS in the immune response has been well established, and it has been reported that both systems communicate with each other (Saper et al., 2012; Petito et al., 2016). Numerous neural and non-neural communication pathways are frequently used to inform central autonomic neural networks of peripheral immune status. These include the microglia preset within the CNS, which belong to the macrophage family, some CNS mediators, such as adrenaline, which has been isolated in the lymphoid organs, and the vagus nerve, which has an important role in the control of systemic inflammation. Kenney and Ganta (2014) reported that vagal paraganglia show chemosensitive assets and that the IL-1β receptors are present on paraganglia located in the abdominal region (Bertozzi et al., 2017). Mac Grory et al. (2010) recently demonstrated that vagal paraganglia play an important role facilitating communication regarding peripheral and central levels of pro-inflammatory cytokines. Another way of communication between the CNS and the immune system is via cytokines that exercise control over mediators present in the CNS, such as in the case of fever (Zhang and An, 2007). IL-1β is involved in various physiological responses of the acute-phase reaction. IL-6 and TNF-α control various centrally mediated physiological responses (Evans et al., 2015). As previously described, PGE2 acts on central neural inflammatory responses, playing a pivot role as effector molecule. (Kenney and Ganta, 2014). Pro-inflammatory cytokines induce COX-2 expression in several central neural sites, which include the blood-brain barrier microvasculature and endothelial cells. The latter induces production of PGE2 (Shih et al., 2015).

In addition to the mechanism listed above, the nervous system is also involved in immunity through the production of neuropeptides such as orexin-A/hypocretin-1 (Sakurai, 2007; Monda et al., 2018).

### Orexin-A in Immunity

The orexin-A (also named hypocretin-1) is a neuropeptide produced in the lateral hypothalamus by definite neurons (Inutsuka and Yamanaka, 2013; Monda et al., 2017). In a particular state of fasting stress, this neuropeptide plays a critical role the peripheral energy balance and the CNS mechanisms: in this manner, it can coordinate sleep-wakefulness and motivated behaviors such as food-seeking (Tsujino and Sakurai, 2009). Orexin-A exerts pleiotropic functions by binding its receptor, orexin type 1 receptor (OX1R), identified on various tissues such as intestine, pancreas, adrenals, kidney, AT, and the reproductive tract (Messina et al., 2016). Orexin-A can be considered as a “multi-tasking” molecule that regulates a set of vital body functions. Such function includes sleep/wake states, eating behavior, reward systems, energy homeostasis, cognition, and mood. Given its importance, a dysfunction of the orexinergic system may be present in various pathological conditions (Chieffi et al., 2017). Interestingly, this neuropeptide has an important role in hippocampal neurogenesis, improving spatial learning and memory abilities. As previously described, orexin deficiency has been reported in several diseases, such as depression, learning, and memory deficits (Zhao et al., 2014). On the other hand, like adiponectin, orexin-A has important roles in energy balance and obesity, and therefore on the accumulation of AT. It is reported that orexin-A influence lipolysis in white fat and thermogenesis in brown fat, affecting the overall energy balance (Hara et al., 2005; Perez-Leighton et al., 2014).

Recently, another role of the orexin-A in inflammation has been recognized. Expression of orexin-A in rats exposed to ischemia/reperfusion was reported to be very high in the stomach, lung, and kidney (Tan et al., 2015). Since inflammation is consequential to the immune response, orexin-A is also involved in immunity (Hara et al., 2005). Fontana et al. (2010) support the hypothesis that narcolepsy is an immune-mediated disease and that the autoantibodies may lead to alterations in the orexin-A system. The loss of orexin-A neurotransmission in narcolepsy may be due to either the spoiled neuronal production and/or secretion of orexin-A, or due to autoantibodies-induced structural damaged to orexin-A producing neurons. During these processes, there is an activation of microglia and macrophages which leads to the release of neurotoxic molecules such as quinolinic acid; these consecutive events can conduct to selective destruction of orexin-A neurons in the hypothalamus (Fontana et al., 2010). Tanaka et al. (2016) have conducted an experimental study in animal model analyzing the alterations in the hypothalamic vigilance system and in the hypothalamic expression of inflammatory factors that occurred after lipopolysaccharide (LPS) administration. They reported that peripheral immune challenge with LPS affected the hypothalamic immune response and vigilance states (Tanaka et al., 2016). This response was altered by the loss of orexin-A. Considering this fact, orexin-A has an important role in metabolic disorders, inflammation, and in the immune response.

## Conclusion

Here, we reported that recent data on adiponectin and orexin-A provide bases for a potential link between AT and CNS. Both the nervous system and AT actively communicate to the immune system through the production of chemical mediators. In particular, adiponectin and orexin-A are two hormones that play crucial roles in the immune system. Adiponectin is actively involved in the immune response by regulating both macrophage proliferation and polarization acts on monocytes and reduces T cells responsiveness, and B cells lymphopoiesis. On the other hand, orexin A is involved in both inflammatory and immune processes. In particular, this peptide is believed to be responsible for the autoantibodies induced damage to neural cells. Skrzypski et al. (2011) have demonstrated through an “*in vivo* study” a direct link between adiponectin and orexin-A. Particularly, their findings suggested that orexin-A stimulates glucose uptake in adipocytes, increasing lipogenesis, inhibiting lipolysis, and stimulating the secretion of adiponectin (Skrzypski et al., 2011). Moreover, it is well described a direct action of orexin-A on peroxisome proliferator-activated receptor γ (PPARγ), blocking its action. As well-described PPARγ plays a critical role in controlling immune and inflammatory responses: in fact, several studies suggested an important role as therapeutic target in several diseases (Pershadsingh, 2004; Ohshima et al., 2012). This could explain the pro-inflammatory action both for orexin A and adiponectin.

The current knowledge strongly encourages further research on the potential functional mechanisms through which adiponectin and orexin-A exert their different actions in the immune system. Further studies are needed to clarify the molecular mechanism and the potential functional interplay between these two mediators.

## Author Contributions

RP, EN, AM, AD, and GM conceived the study and participated in its design. RP, MLM, VM, GC, AV, AD, and GM contributed to the conception and design. OS, EP, CZ, DP, and GM wrote the manuscript. MM, IC, VM, and RP drafted the article and revised it critically for important intellectual content. RP, EN, GM, and AD performed the final approval of the version to be published. All the authors read and approved the final manuscript.

## Conflict of Interest Statement

The authors declare that the research was conducted in the absence of any commercial or financial relationships that could be construed as a potential conflict of interest.
